# Preparation and Characterization of Electromagnetic-Induced Rupture Microcapsules for Self-Repairing Mortars

**DOI:** 10.3390/ma15103608

**Published:** 2022-05-18

**Authors:** Erwang Li, Wei Du, Ronghua Zhuang, Mingfang Ba, Lianwang Yuan, Qian Zhang, Yuepin Zhang

**Affiliations:** 1 School of Material Science and Chemical Engineering, Ningbo University, Ningbo 315211, China; erwang_li@163.com (E.L.); zhangyuepin@nbu.edu.cn (Y.Z.); 2 State Key Laboratory of Silicate Materials for Architectures, Wuhan University of Technology, Wuhan 430070, China; rhzhuang@whut.edu.cn; 3 School of Civil and Environmental Engineering, Ningbo University, Ningbo 315211, China; bamingfang@nbu.edu.cn; 4 Shandong Provincal Key Laboratory of Preparation and Measurement of Building Materials, University of Jinan, Jinan 250022, China; lwyuan0910@126.com (L.Y.); qian.zh@hotmail.com (Q.Z.)

**Keywords:** self-repairing, electromagnetic-induced rupture microcapsules, Fe_3_O_4_ nano-particles, cracks, ultrasonic testing

## Abstract

Cement-based materials are susceptible to internal cracks during service, leading to a reduction in their durability. Microcapsules can effectively self-repair cracks in cement-based materials. In this study, novel electromagnetic-induced rupture microcapsules (DWMs) were prepared by using the melt dispersion method with Fe_3_O_4_ nano-particles/polyethylene wax as the shell and epoxy resin as the repairing agent. The core fraction, compactness, particle size distribution, morphology, and chemical structure of DWMs were characterized. DWMs were subsequently incorporated into the mortar to measure the pore size distribution, compressive strength recovery, and maximum amplitudes of the pre-damaged mortar after self-repairing. DWMs were also evaluated for their ability to self-repair cracks on mortar surfaces. The results showed that the core fraction, remaining weight (30 days), and mean size of DWMs were 72.5%, 97.6 g, and 220 μm, respectively. SEM showed that the DWMs were regular spherical with a rough surface and could form a good bond with cement matrix. FTIR indicated that the epoxy resin was successfully encapsulated in the Fe_3_O_4_ nano-particles/polyethylene wax. After 15 days of self-repairing, the harmful pore ratio, compressive strength recovery, and maximum amplitude of the pre-damaged mortars were 48.97%, 91.9%, and 24.03 mV, respectively. The mortar with an initial crack width of 0.4–0.5 mm was self-repaired within 7 days. This indicated that the incorporation of DWMs can improve the self-repair ability of the mortar. This work is expected to provide new insights to address the mechanism of microcapsule rupture in self-repairing cement-based materials.

## 1. Introduction

Concrete is the second most consumed substance in the world after water [1]. It can be found almost anywhere in human existence. However, due to their brittle characteristics, concrete structures are prone to damage, especially cracks, under the influence of harsh environments, external loads, or fire [2,3,4,5]. The appearance and expansion of cracks can have a negative impact on the mechanical properties and durability of cement-based materials [6]. Therefore, inspection and maintenance have become an important part of concrete structures [7,8]. However, long-term maintenance is very difficult due to the large amount of labor and financial support required [9,10]. Also, internal cracks in the concrete are not easily inspected and repaired in a timely manner [11]. Therefore, the research of self-repairing materials for cracks in cement-based materials has attracted considerable attention [12,13,14].

In numerous studies, the self-repairing of cracks is illustrated as a complex combination of physical and chemical reactions [15,16,17]. There are several reasons for this phenomenon, such as the formation of calcium carbonate [18], impurities in the water [19], the further hydration of cement particles, and the expansion of hydration products [20]. In addition, mineral admixtures [21], microorganisms [22], shape memory alloys [23], and microcapsules [24] can also accelerate the self-repairing process when added to cement-based materials. Compared with other self-repairing methods, microcapsules have the advantages of high environmental adaptability and fast repairing speed, which are suitable attributes for application [25,26].

The microcapsule self-repairing method was first proposed by White in 2001 [27]. Microcapsules contain a core material (repairing agent) that is encapsulated in a shell material that will rupture due to stress concentration when a crack occurs and expands. Due to the capillary action, the repairing agent is released and flows into the crack. Solidification then occurs upon contact with the curing agent to repair the crack and prevent further expansion. Litina [28] prepared microcapsules using gelatin and gum Arabic cross-linked to form a shell encapsulated with mineral oil and liquid sodium silicate solution, which can effectively improve the mechanical properties of mortars. Han [29] prepared microcapsules using urea—formaldehyde resin coated with epoxy resin, and the results showed that incorporating the microcapsules into cement-based materials substantially improved their permeability. However, microcapsules are difficult to rupture in time due to the high mechanical strength of the thermoset polymer shell [30]. To solve this problem, we used microcrystalline wax to encapsulate epoxy resin to prepare microcapsules in our previous experiments [31]. The results showed that these microcapsules have good self-repairing ability for the mechanical properties and surface cracks of cement-based materials. However, the mechanical properties of these microcapsule shell materials are relatively low and there is a high risk of rupture during the concrete mixing process. In order to improve the strength of microcapsules, some researchers have synthesized the shell materials by adding nanomaterials. Yang [32] added nano-CaCO_3_ particles into melamine—formaldehyde resin as shell material to coat aromatic oil rejuvenating agent. Du [33] applied a nano-CaCO_3_/ceresin wax composite shell containing epoxy resin to prepare the microcapsules.

However, the microcapsules are not always located at the tip of the crack. When cracks appear inside the cement-based materials, the microcapsules may not rupture if they are not at the tip of the crack, so the crack cannot be repaired. To solve this problem, new microcapsule rupture trigger mechanisms need to be developed. Yang [34] demonstrated that self-repairing of electrical damage in thermoplastic polymers using superparamagnetic nanoparticles under the stimulation of an external oscillating magnetic field can lead to appropriate repair results. Hu [35] prepared a magnetically sensitive microcapsule using Fe_3_O_4_/polyallylamine polyelectrolyte for constructing a shell in drug delivery. The above studies provide the idea of adding magnetic nanomaterials to the shell material to prepare microcapsules that can be stimulated by electromagnetic fields that cause the shell to melt and rupture due to increased temperature.

This paper aims to improve the self-repairing ability of microcapsules for mortars. Electromagnetic-induced rupture microcapsule (DWMs) with Fe_3_O_4_ nano-particles and polyethylene wax as the shell, and epoxy resin as the repairing agent, were prepared using a melt dispersion method. The core fraction, compactness, particle size distribution, morphology, and chemical structure of the DWMs were characterized. The DWMs were subsequently incorporated into the mortar, and the pore size distribution, compressive strength recovery, and maximum amplitude of the pre-damaged mortars after self-repairing were measured. The self-repairing ability of DWMs on mortar surface cracks was evaluated.

## 2. Materials and Methods

### 2.1. Materials

Polyethylene wax (melting point: 110 °C) was obtained from Sinopharm Chemical Reagent Co., Ltd. (Shanghai, China). The Fe_3_O_4_ nano-particles (particle size: 50 nm) was supplied from Nangong Xindun alloy welding material spraying Co., Ltd. (Xingtai, China). Epoxy resin, 2-ethyl-4-methylimidazole and N, N-dimethylformamide (DMF) were purchased from Chengdu Micxy Chemical Co., Ltd. (Chengdu, China) Perfluorotributylamine was provided by Beijing Letai Chemical Reagent Co., Ltd. (Beijing, China) Ordinary Portland cement (CEMI 42.5N) was bought from Huaxin Cement Co., Ltd. Sand (modulus: 2.35) was applied by Ningbo Pulunxiang building materials Co., Ltd (Ningbo, China).

### 2.2. Preparation of Microcapsules

**Figure 1:**
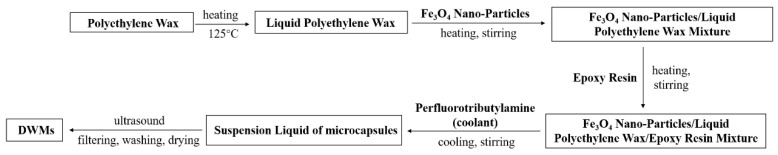
Preparation procedure of DWMs.

The microcapsules were prepared using the melt dispersion method, which is a physical method with a simple process, high preparation efficiency, and no chemical reactions during the preparation [33]. At first, the raw materials for shells (1. polyethylene wax; 2. Fe_3_O_4_ nano-particles/polyethylene wax) were melted and dispersed in a three-neck flask using an oil bath. Then the epoxy resin diluted with N, N-dimethylformamide (DMF) was added to the mixture of the shell with continuous heating and stirring, where the amount of DMF was 15% of the weight of epoxy resin. Next, perfluorotributylamine (coolant) was poured into the mixture so that the temperature of the mixture was rapidly reduced to below the melting point of polyethylene wax to obtain the microcapsule suspension. The suspension was poured into a beaker and shaken with ultrasound for 30 min. Finally, the microcapsules were filtered to obtain the finished products. The preparation parameters of the microcapsules are indicated in Table 1. Figure 1 describes the preparation procedure of the microcapsules (DWMs).

### 2.3. Preparation of Mortars

The mix designs of mortars were shown in Table 2. First, cement, sand, and microcapsules were mixed in a mortar mixer for 1 min, then water and 2-ethyl-4-methylimidazole were added to the mortar mixer and mixing was continued for 2 min. 2-ethyl-4-methylimidazole was added at 20% of the weight of the diluted epoxy resin. Prismatic samples with casting dimensions of 40mm × 40mm × 160mm were used for mechanical property testing [31]. Finally, the mortars were demolded and placed in a standard curing room (20 ± 2 °C, 95%RH) for 28 days. The microcapsules used for mortar preparation were left in air for more than 30 days.

### 2.4. Measurement and Characterization

#### 2.4.1. Core Fraction

To measure the core fraction of the microcapsules, some microcapsules were randomly selected and weighed. These microcapsules were then ground to allow all of the epoxy resin to flow out, filter, and dry, and the remaining residue was then weighed [36]. The core fraction of the microcapsules was measured through Equation (1).

(1) $$\tau =\frac{\tau_{1}-\tau_{2}}{\tau_{1}}\times 100\%$$

where *τ* is the microcapsule core fraction (%), *τ*_1_ is the initial weight of microcapsules (g), and *τ*_2_ is the weight of residue (g).

#### 2.4.2. Compactness of Microcapsules

At first, 100 g of microcapsules were weighed. These microcapsules were then moved to a box (20 ± 2 °C, 50% RH) for storage. The compactness of the microcapsules was assessed by weighing the remaining weight of the microcapsules after 1, 2, 3, 4, 5, 6, 7, 15, and 30 days, respectively.

#### 2.4.3. Size Distributions

The mean size and size distribution of the microcapsules were determined using a laser particle size analyzer (Mastersizer 2000, Malvern Instruments Ltd., Malvern, England). Before the test, the microcapsules were placed in a drying box at 40 °C for 24 h. Then, 1 g microcapsules were dispersed by 50 mL deionized water in the analysis box. During the laser diffraction measurement, the particles were passed through a focused laser beam. These particles scatter light at an angle that was inversely proportional to their sizes. The angular intensity of the scattered light was measured via a series of photosensitive detectors. The size distribution of the after-sieving microcapsules was determined from the OM images of the microcapsules using a commercial dimensional measurement software.

#### 2.4.4. Morphology

The morphologies of the microcapsules were observed using scanning electron microscopy (SEM) (Hitachi S-4800, Tokyo, Japan). Before their observation, the microcapsules were spread uniformly on conductive adhesive tape and a thin layer of gold was sputtered on their surface. The SEM images were taken at a magnification of 200×.

#### 2.4.5. Fourier Transform Infrared Spectroscopy (FTIR)

A Fourier transform infrared spectrometer (Nexus, Thermo Nicolet Corporation, Madison, WI, USA) was used to characterize microcapsules. The Fe_3_O_4_ nano-particles, polyethylene wax, and microcapsules with KBr were compressed into slices after being mixed and ground, which were then measured through the FTIR. The epoxy resin and DMF were tested after it was brushed on the KBr tablets. With a resolution for 4 cm^−1^, the FTIR spectra were recorded within the range of 400–4000 cm^−1^ in absorption mode.

#### 2.4.6. Self-Repairing of Pre-Damaged Mortars

**Figure 2:**
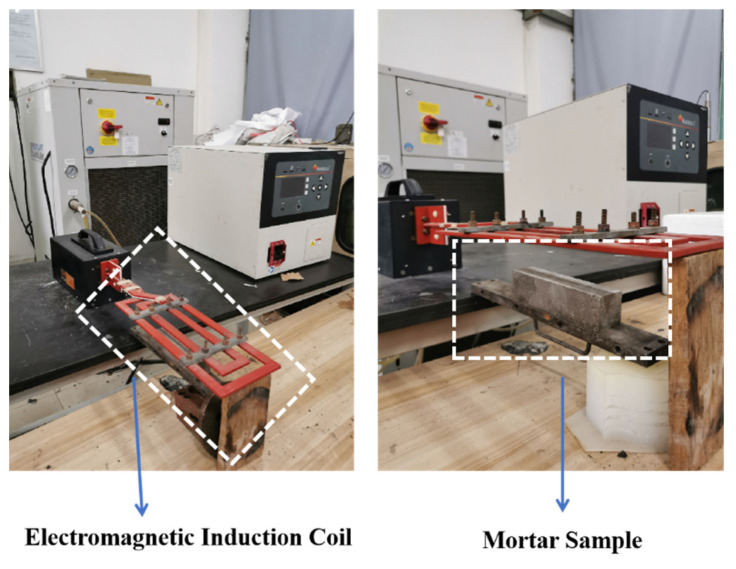
Electromagnetic induction heating system.

After 28 days of standard curing, the flexural and compressive strengths of the mortars were measured, which were expressed by f_1_ and f_2_, respectively. Take three samples from each group to calculate the average flexural strength, and take six samples for mortars compressive strength test. In order to obtain pre-damaged mortar samples, the mortar was pre-loaded to 60% f_2_. After that, SJ-0 and SJ-1 were placed at room temperature for 3, 9, and 15 days. SJ-2 was heated under an electromagnetic field (Figure 2) for 45 min with a heating interval of 30 s, and then left at room temperature for 3, 9, and 15 days. Where the distance between the heating coil and the upper surface of SJ-2 is 0.5 cm, the frequency of the electromagnetic induction heater is 124 kHz, and the output voltage of the electromagnetic induction heating equipment is 600 V. Finally, the samples were reloaded and the compressive strength recovery of the mortar was measured according to Equation (2).

(2) $$\sigma =\frac{f_{2}{}^{\prime }}{f_{2}}\times 100\%$$

where *σ* is the compressive strength recovery of the mortar, *f*_2_ is the initial compressive strength of the mortar, and *f*_2_′ is the compressive strength of the mortar after self-repairing.

#### 2.4.7. Pore Size Distribution

The nuclear magnetic resonance (NMR) spectrometer (MesoMR25, Suzhou Newman Analytical Instrument Co., Ltd., Suzhou, China) was used to measure pore size distribution of the mortar. The measuring principle of pore size distribution by NMR technique is that the water in the pore will resonate with the molecule under low power magnetic field, and relaxation occurs during the process of energy exchange and release, and the relaxation time can reflect the pore size. NMR is suitable for measuring a pore size distribution of 2 nm–1 mm, which has been applied to the study of the pore structure of the mortar. The size of the mortar used for NMR test was 25 mm × 25 mm × 15 mm. Prior to the test, the mortar sample was wiped to clean the surface powder and was vacuum-saturated with water for 24 h and then stored in water for another week. The resonance frequency was 23.40 MHz, the temperature of the magnet was 32.00 ± 0.02 °C, and the instrument probe diameter was 25 mm. The pore diameter was computed using Equation (3).

(3) $$\frac{1}{T_{2}}=\rho (\frac{S}{V}{)}_{pore}$$

where *T*_2_ is the relaxation time of water in the pore (ms), *ρ* is the surface relaxation rate (70 μm/ms), and (*S*/*V*)*_pore_* is the pore surface area to volume ratio.

#### 2.4.8. Ultrasonic Testing

The pore size distribution and mechanical properties have a great impact on the quality of cement-based materials. If these properties do not meet the requirements and thus lead to accidents, they can cause huge losses of people and property. At present, tests of macroscopic mechanical properties and the microscopic pore size distribution of mortars are carried out by destroying samples. However, in engineering, we need to evaluate the service performance of buildings without damaging them, so the non-destructive testing method represented by ultrasonic testing technology is particularly important.

A generator (AFG3022C, Tektronix Co., Ltd., Shanghai, China) was used to generate and transmit ultrasonic waves. An oscilloscope (MDO 3024, Tektronix Co., Ltd., Shanghai, China) was applied to receive and characterize the ultrasonic waves. The ultrasonic frequency was 107 kHz and the voltage was controlled at ±5 V. The radial piezoelectric ultrasonic transducer emitted ultrasonic waves when collecting the data. The standard transducers were mounted on both sides of the sample and the coupling agent used was petroleum jelly. During the test, the data and images were automatically connected to a computer. Finally, the ultrasonic signal was converted from the time domain to the frequency domain using the Fast Fourier Transform (FFT) function of the software [37]. Ultrasound can propagate in different media with highly repeatable waveforms and frequencies when propagating at a steady state of energy. The mortars were tested before and after 15 days of self-repairing.

#### 2.4.9. Surface Cracks Self-Repairing

**Figure 3:**
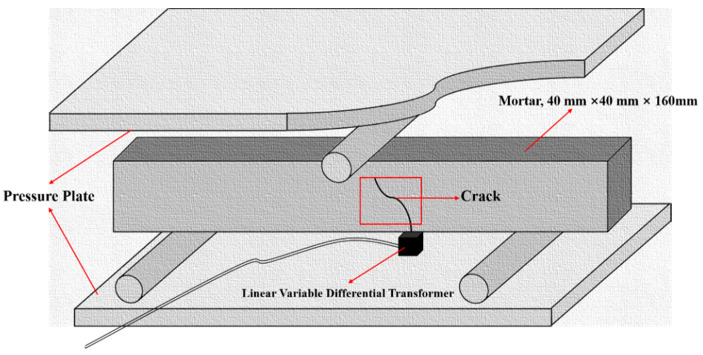
Schematic diagram of pre-cracked mortar by the three-point bending method [31].

After 28 days of standard curing, the mortar was pre-cracked by the three-point bending method (Figure 3). The crack widths of SJ-1 and SJ-2 before and after 1, 2, 3, 5, 7, and 15 days of self-repairing in the laboratory or electromagnetic induction heating system were measured by the crack tester (PES-30, Botte Instrument Co., Ltd., Lianyungang, China). For surface cracks of mortars, it is the most intuitive method for obtaining crack images directly and detecting them. The number of pixels included in the crack image before and after the self-repairing of the cracked sample was calculated using image analysis software, which is to say that the area of the crack with different self-repairing time, and the repairing ratio of the crack area was calculated according to the Equation (4).

(4) $$\eta_{crack}=\frac{A_{0}{-A}_{t}}{A_{0}}\times 100\%$$

where *η_crack_* is the repairing ratio of the crack area (%); *A*_0_ is the number of initial crack pixels, *A_t_* is the number of crack pixels after self-repairing.

## 3. Results and Discussion

### 3.1. Core Fraction

The core fractions of the polyethylene wax with an epoxy resin microcapsule (SWMs), and Fe_3_O_4_ nano-particles/polyethylene wax with an epoxy resin microcapsule (DWMs), are shown in Table 3. It was found that the core fractions of the SWMs and DWMs were 64.9% and 72.5%, respectively. This is because SWMs and DWMs have different viscosities of the shell material. For SWMs, there is only one shell material, namely polyethylene wax, which has a low viscosity and can be evenly dispersed after heating and melting. After adding the coolant, the particle size of the SWMs is smaller, resulting in a small amount of epoxy resin remaining outside the shell, and therefore the core fraction of SWMs is low. The addition of Fe_3_O_4_ nano-particles to the polyethylene wax increased the viscosity of the shell material mixture, decreased the dispersion of the material, reduced the solidification time of the shell, and improved the ability of the shell material to encapsulate epoxy resin, resulting in a higher core fraction of DWMs.

### 3.2. Compactness

**Figure 4:**
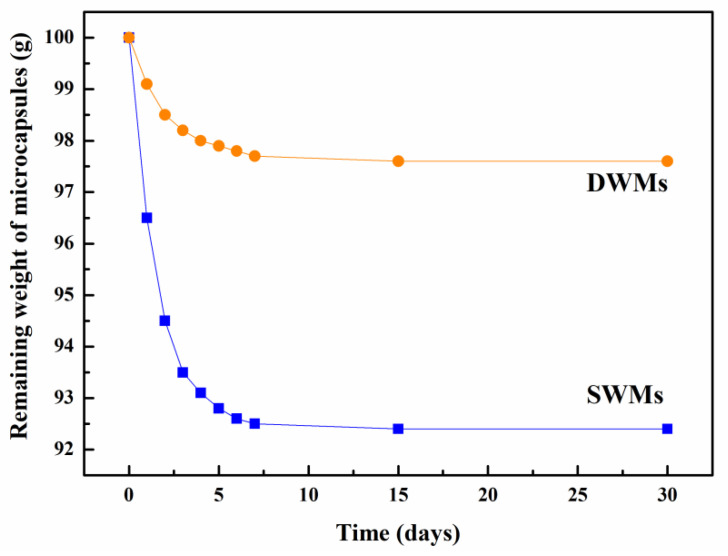
Remaining weight of SWMs and DWMs.

Figure 4 depicts the changes in the remaining weights of SWMs and DWMs after placing them for different days. As can be seen from Figure 4, the remaining weights of the SWMs and DWMs decreased significantly from the first day of preparation to the seventh day, but then leveled off. After 15 days of placement, the remaining weights of the SWMs and DWMs no longer changed. After 30 days, the remaining weight of the SWMs was 92.4 g, which was 7.6% lower than the initial value, while the remaining weight of the DWMs was 97.6 g, which was only 2.4% lower than the initial value. This was because the addition of Fe_3_O_4_ nano-particles to the polyethylene wax filled the micro-defects on the surface of the DWMs (Fe_3_O_4_ nano-particles/polyethylene wax) shell and made the structure more compact, thus improving its storage capacity for epoxy resin and reducing its weight loss.

### 3.3. Particle Size Distributions

**Figure 5:**
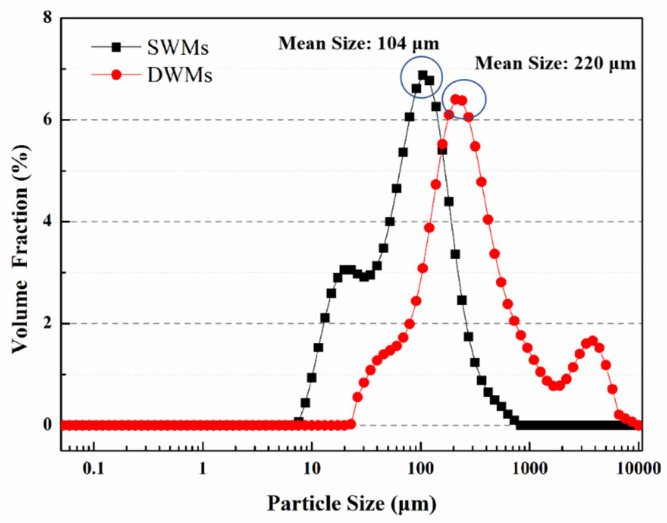
Particle size distributions of SWMs and DWMs.

Figure 5 shows the particle size distribution of the SWMs and DWMs. The particle size distribution of the SWMs mainly ranges from 10 μm to 300 μm, and the mean size is 104 μm. The particle size distribution of the DWMs mainly ranges from 90 μm to 700 μm, and the mean size is 220 μm. By comparing the two microcapsules, we can find that the particle size of the SWMs is significantly smaller than that of DWMs. This is due to the low viscosity of polyethylene wax, which can be evenly dispersed under heating and stirring conditions. After adding the coolant, the solidification time of polyethylene wax is short, and the SWMs can form small particle size rapidly under the action of shear force. After the addition of Fe_3_O_4_ nano-particles, the viscosity of the DWMs shell material mixture increases significantly, its dispersibility decreases accordingly, the required cooling solidification time is prolonged, and the particle size of DWMs increases at a constant stirring rate.

### 3.4. Morphology

**Figure 6:**
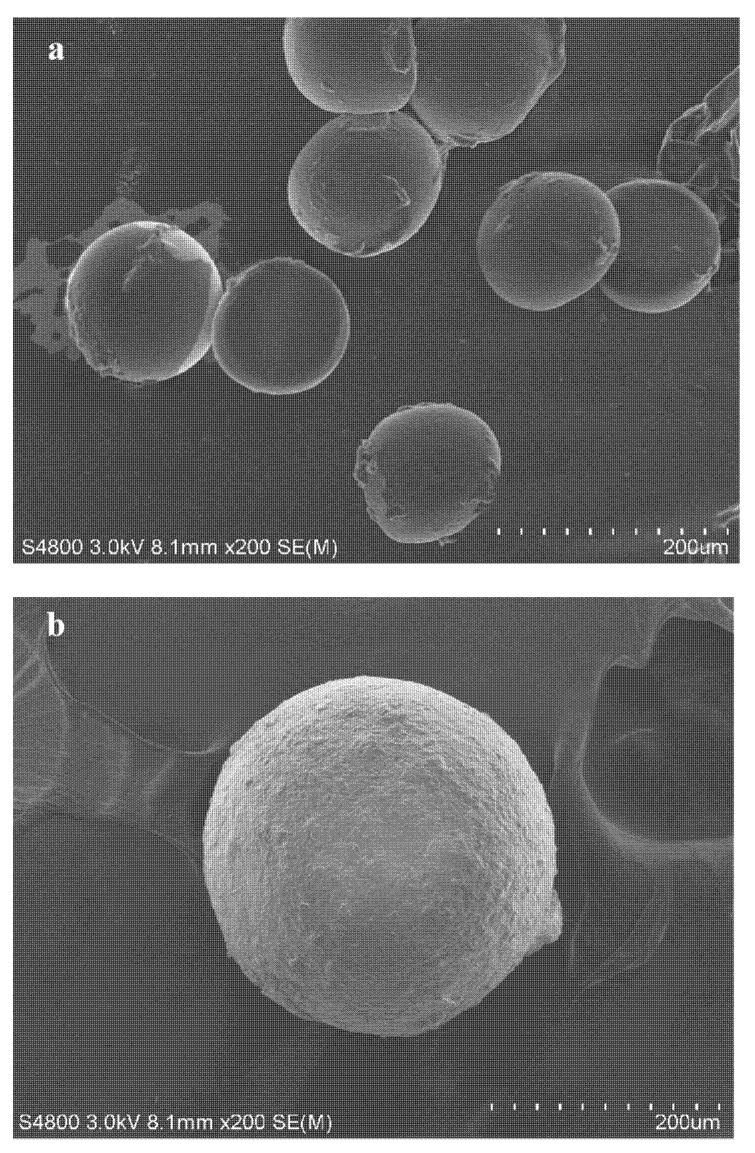
SEM image of microcapsules. (**a**) SWMs, (**b**) DWMs.

The surface morphology of microcapsules is shown in Figure 6. As shown in Figure 6a, the SWMs are regularly spherical with a particle size distribution between 85–120 μm. As can be seen from Figure 6b, the surface of DWMs is rougher with a particle size of about 220 μm. This is because after mixing the Fe_3_O_4_ nano-particles with polyethylene wax, the viscosity of the mixture (shell material) increases and the dispersibility decreases, resulting in a larger particle size and a relatively rougher surface of the microcapsules. With the increase of microcapsule particle size, more repairing agent can be stored to improve the self-repairing effect at a later stage, and the rough surface of the microcapsules can be more conducive to forming a suitable bond between their shell and cement matrix.

### 3.5. FTIR Analysis

**Figure 7:**
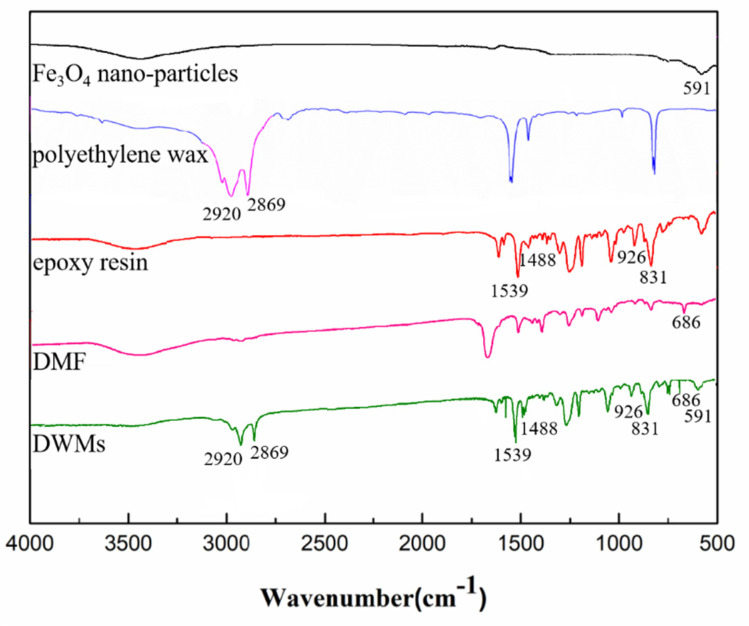
FTIR spectra of Fe_3_O_4_ nano-particles, polyethylene wax, and epoxy resin, DMF and DWMs.

The FTIR spectra of Fe_3_O_4_ nano-particles, polyethylene wax, epoxy resin, DMF, and DWMs are shown in Figure 7. The peaks at 2920 cm^−1^ and 2869 cm^−1^ are attributed to the symmetric and asymmetric stretching vibrations of the –CH_2_– and –CH_3_ groups in the polyethylene wax, respectively, while the absorption peaks at 1539 cm^−1^ and 1488 cm^−1^ are due to the stretching vibration of aromatic C–C and the symmetric bending of dimethyl in epoxy resin. The absorption peaks observed at 926 cm^−1^ and 831 cm^−1^ are the characteristic absorption peaks of C–O and C–O–C in the ethylene oxide moiety, respectively. In addition, the peak at 591 cm^−1^ is a characteristic absorption peak of Fe_3_O_4_ nano-particles, while an absorption peak belonging to the characteristic peak of the amide I band is found at 686 cm^−1^, indicating that a small amount of DMF is encapsulated in DWMs. The characteristic peaks of Fe_3_O_4_ nano-particles, polyethylene wax, epoxy resin, and DMF can be seen in the FTIR spectra of DWMs, which proves that the epoxy resin is successfully encapsulated in the shell of polyethylene wax and Fe_3_O_4_ nano-particles.

### 3.6. Pore Size Distribution

**Figure 8:**
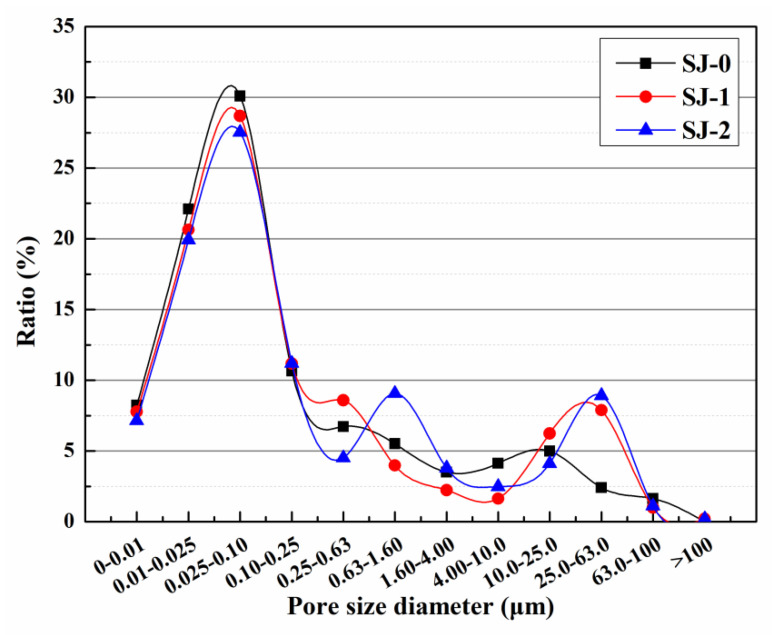
Pore size distribution of mortars.

The initial pore size distributions of mortars are shown in Figure 8. Pores with diameters greater than 0.1 μm in the mortar reduce its mechanical properties, and these pores are called harmful pores [38]. The harmful pore ratios of SJ-0, SJ-1, and SJ-2 were 39.57%, 42.92%, and 45.41%, respectively. Compared to SJ-0, the harmful pore ratios of SJ-1 and SJ-2 increased by 3.35% and 5.84%, respectively. This may be due to the larger size of the SWMs and DWMs, which increases the internal pore size of the mortar and reduces the compactness of the mortar, thus increasing the harmful pore ratio of the mortar [39].

**Figure 9:**
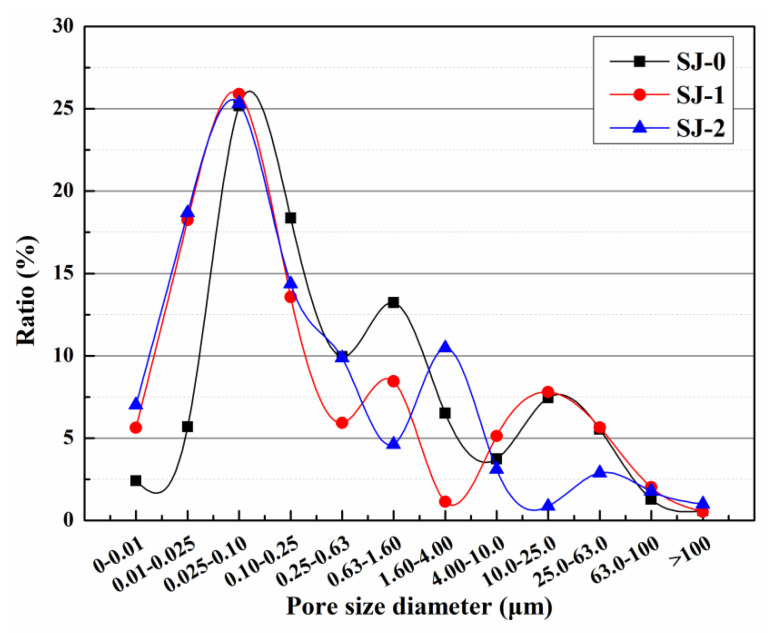
Pore size distribution of mortars after self-repairing.

**Figure 10:**
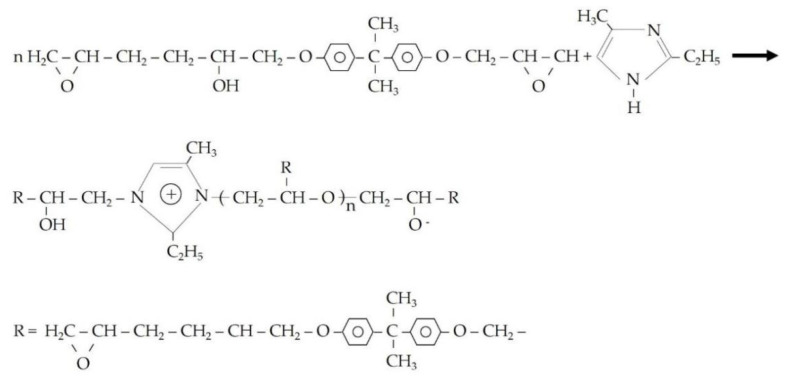
Chemical reaction process of epoxy resin and 2-ethyl-4-methylimidazole.

Figure 9 shows that after 15 days of self-repairing, the harmful pore ratios of pre-damaged SJ-0, SJ-1, and SJ-2 were 66.72%, 50.23%, and 48.97%, respectively. Compared with the initial harmful pore ratio, the harmful pore ratios of SJ-0, SJ-1, and SJ-2 increased by 27.15%, 7.31%, and 3.56%, respectively. The results showed that the harmful pore ratios of SJ-1 and SJ-2 were significantly lower and close to the initial values after self-repairing compared to SJ-0. This is because mortar produces internal cracks after pre-damage and the cracks encounter microcapsules during expansion. The microcapsules are prone to rupture under the crack tip stress, and the released epoxy resin (repairing agent) cures in contact with 2-ethyl-4-methylimidazole (curing agent), and the generated repairing products are used to fill the cracks, thus reducing the harmful pore ratios in the mortar. However, there is no guarantee that microcapsules will be present in the crack extension track. If this occurs, the microcapsules may not rupture, resulting in cracks that cannot repair. In contrast, SJ-2 is doped with DWMs, which are microcapsules with Fe_3_O_4_ nano-particles on the surface. Fe_3_O_4_ nano-particles have excellent magnetic permeability, which helps to generate more heat in the electromagnetic field to reduce hysteresis loss. In addition, Fe_3_O_4_ nano-particles are a ferrimagnet that generates Joule heat due to eddy current loss. Under the action of the applied electromagnetic field, the temperature of the shell material rises and melts, and the epoxy resin encapsulated inside flows out to contact with 2-ethyl-4-methylimidazole and reacts to fill the cracks, which enhances the self-repairing ability of the microcapsules to the cracks and reduces the harmful pore ratios in the mortar. The curing process of epoxy resin and 2-ethyl-4-methylimidazole is shown in Figure 10.

### 3.7. Flexural Strength and Compressive Strength

The flexural strengths and compressive strengths of SJ-0, SJ-1, and SJ-2 are shown in Table 4. The flexural strengths of SJ-0, SJ-1, and SJ-2 were 8.2 MPa, 7.9 MPa, and 7.5 MPa, respectively. Compared with SJ-0, the flexural strengths of SJ-1 and SJ-2 were reduced by 3.7% and 8.5%, respectively. The compressive strengths of SJ-0, SJ-1, and SJ-2 were 33.4 MPa, 32.6 MPa, and 30.9 MPa, respectively. Compared to SJ-0, the compressive strengths of SJ-1 and SJ-2 decreased by 2.3% and 7.5%, respectively. This is due to the large difference in Young’s modulus between the cement matrix and the shell of the microcapsule. When microcapsules are added to the cement mortar, there will be gaps at the joints between the microcapsules and the cement matrix, which will increase the pores in the matrix material and reduce the stiffness, flexural strength, and compressive strength of the mortar [40]. In addition, the addition of microcapsules to the mortar will also reduce the compactness of the mortar during the molding process, and affect the particle gradation of the mortar, thereby reducing the flexural strength and compressive strength of the mortar [41]. The compressive strength of SJ-2 is relatively low because the particle size of DWMs is larger than that of SWMs, which is consistent with the results of the pore size distribution tests in Section 3.6.

### 3.8. Compressive Strength Recovery

**Figure 11:**
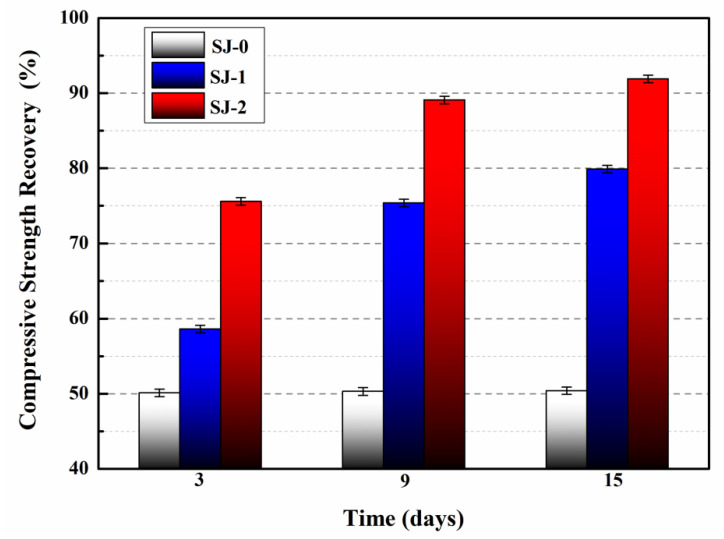
Compressive strength recovery of mortars.

The compressive strength recovery of the mortars is illustrated in Figure 11. The compressive strength recovery of the SJ-0 was 50.1%, 50.3%, and 50.4% at 3, 9, and 15 days, respectively. The results indicate that the inherent self-repairing ability of the cementitious material cannot repair the pre-damaged SJ-0.

In comparison, after 3 days of self-repairing, the compressive strength recovery of SJ-1 and SJ-2 were 58.6% and 75.6%, respectively. When the self-repairing time of SJ-1 and SJ-2 reached 15 days, their compressive strength recovery were 79.9% and 91.9%, respectively, indicating that the compressive strength recovery of SJ-1 and SJ-2 were significantly improved. This is because SJ-1 develops internal cracks after being pre-damaged by 60% f_2_, and these cracks will continue to extend over time. The extending stress of the cracks will damage the shell of SWMs embedded in SJ-1, and the epoxy resin originally encapsulated in the SWMs will flow out, contact, and react with 2-ethyl-4-methylimidazole, and the generated repairing product will fill the cracks and recover the compressive strength of SJ-1. However, the path of crack expansion in the mortar after pre-damage is relatively random. If microcapsules are not encountered during crack expansion or if the number of microcapsules is insufficient, the self-repairing ability of the mortar will be compromised. Compared with SJ-1, SJ-2 showed higher recovery of compressive strength at day 3 and day 15, indicating that SJ-2 has faster self-repairing and better mechanical properties recovery. This is due to the addition of DWMs (microcapsules with Fe_3_O_4_ nano-particles on the surface) in SJ-2. Fe_3_O_4_ nano-particles have good magnetic permeability and generate heat in response to external electromagnetic fields, causing the DWMs to rupture. The epoxy resin flows out of the fractured DWMs, comes in contact with 2-ethyl-4-methylimidazole, and reacts to fill the cracks. Compared with the SJ-1 (self-repairing at room temperature), SJ-2 has a higher self-repairing temperature, which makes the reactant molecules (epoxy resin and 2-ethyl-4-methylimidazole) more active and the intermolecular movement is accelerated, thus increasing the curing reaction speed and enhancing the recovery of mortar compressive strength through microcapsules.

### 3.9. Analysis of Ultrasonic Waveform

**Figure 12:**
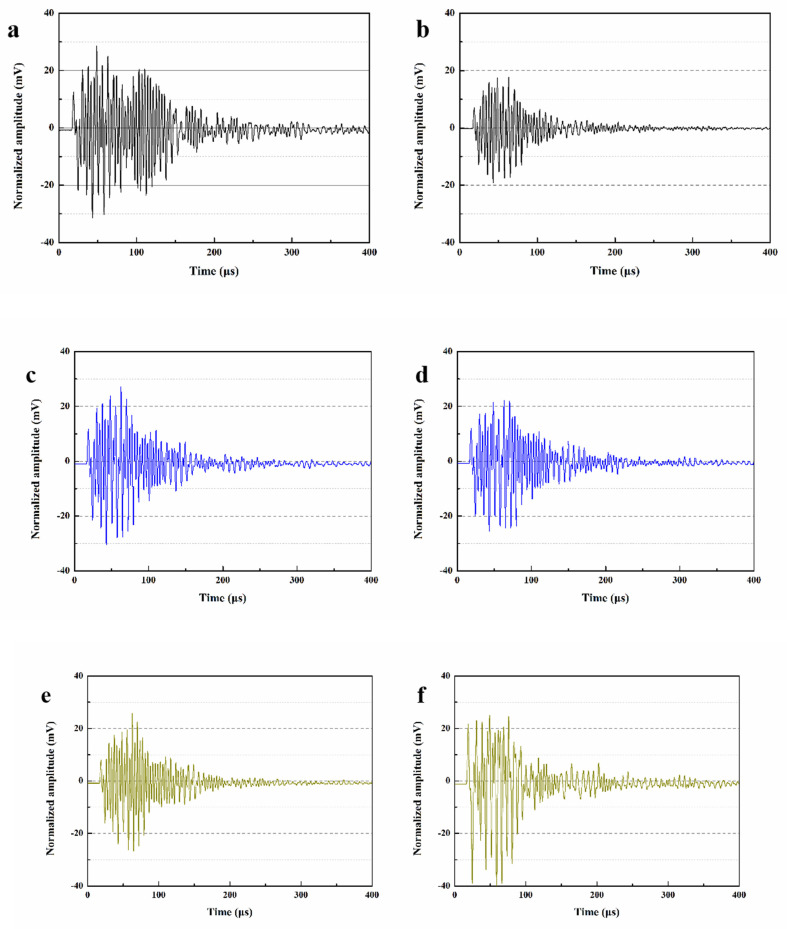
**Ultrasonic waveform of mortars.** (**a**) initial ultrasonic waveform of SJ-0, (**b**) ultrasonic waveform of self-repaired SJ-0, (**c**) initial ultrasonic waveform of SJ-1, (**d**) ultrasonic waveform of self-repaired SJ-1, (**e**) initial ultrasonic waveform of SJ-2, (**f**) ultrasonic waveform of self-repaired SJ-2.

Figure 12 shows the ultrasonic waveforms of different mortars before and after self-repairing. After 28 days of standard curing, the initial maximum amplitudes of SJ-0, SJ-1 and SJ-2 were 28.75 mV, 27.02 mV and 26.21 mV, respectively (Figure 12a,c,e). Compared with SJ-0, the maximum amplitudes of both SJ-1 and SJ-2 decreased slightly. This is because the acoustic impedance of the mortar encountered during ultrasonic wave propagation is much larger than that of air. When pores in the mortar are encountered, the ultrasonic signal attenuates, resulting in a decrease in the amplitude of the received signal [42]. The higher the mortar density, the fewer pores encountered during ultrasonic wave propagation, the smaller the amplitude of signal attenuation, and the larger the wave amplitude. According to Section 3.6, SJ-1 and SJ-2 have a higher ratio of harmful pores compared to SJ-0, and thus their amplitudes are lower.

The maximum amplitudes of SJ-0, SJ-1 and SJ-2 after self-repairing were 18.02 mV, 22.15 mV, and 24.03 mV, respectively (Figure 12b,d,f). The results showed that the maximum amplitude of SJ-0 was significantly lower than the initial value after 15 days of self-repairing, while the maximum amplitude of SJ-1 and SJ-2 recovered significantly. This is due to the fact that defects such as microcracks and macropores appear inside the mortar after being subjected to external stress, and the presence of these defects leads to a weakening of the ultrasonic signal during propagation and a decrease in the maximum amplitude received. There are no microcapsules in SJ-0, and its internal structure cannot be recovered after damage, so its maximum amplitude is minimal. For SJ-1, the SWMs fracture under the stress of the crack tip and release the epoxy resin. The epoxy resin cures in contact with 2-ethyl-4-methylimidazole, which repairs the microcracks and macropores, leading to an increase in the maximum amplitude of SJ-1. In the presence of an external magnetic field, the shell of DWMs melts due to the increase in temperature, and the out-flowing epoxy resin cures in contact with 2-ethyl-4-methylimidazole at high temperature, which can recover the maximum amplitude of SJ-2 to a greater extent.

### 3.10. Parameters Analysis of Received Wave

**Figure 13:**
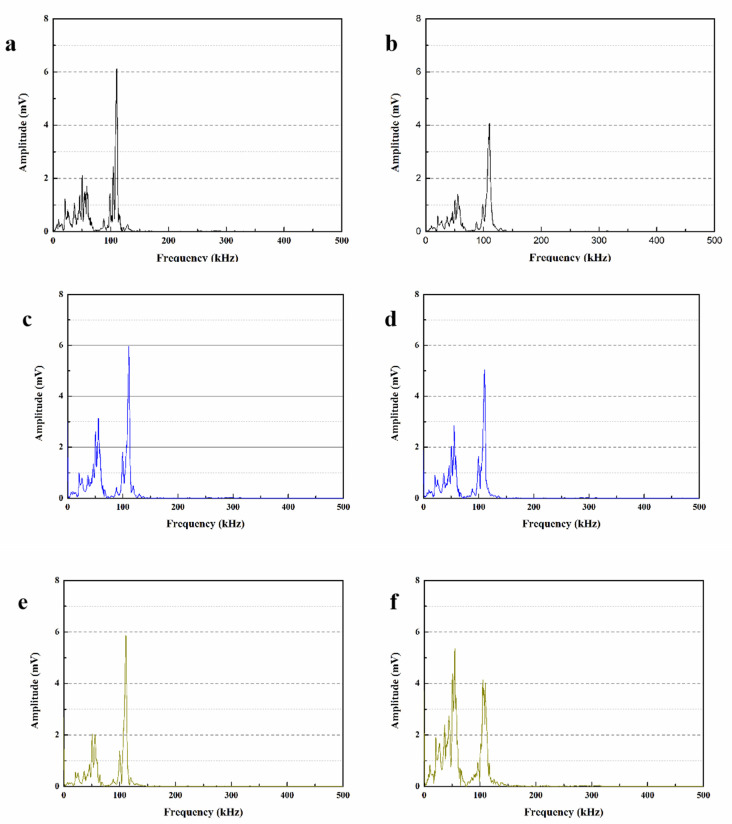
**Ultrasonic frequency of mortars.** (**a**) initial maximum amplitudes of the dominant frequency of SJ-0, (**b**) maximum amplitudes of the dominant frequency of self-repaired SJ-0, (**c**) initial maximum amplitudes of the dominant frequency of SJ-1, (**d**) maximum amplitudes of the dominant frequency of self-repaired SJ-1, (**e**) initial maximum amplitudes of the dominant frequency of SJ-2, (**f**) maximum amplitudes of the dominant frequency of self-repaired SJ-2.

In order to obtain clearer results and estimate the trend of ultrasonic waves, this section uses the fast Fourier transform (FFT) method with Gaussian function to process the ultrasonic wave shape of the mortar in Figure 12, and the obtained ultrasonic wave frequencies are shown in Figure 13. The main frequency tested in this experiment is about 107 kHz, and the peaks shown in the low frequency range are caused by the attenuation of ultrasonic energy due to the presence of air in the mortar pores [43].

The maximum amplitudes of the main frequencies for SJ-0, SJ-1 and SJ-2 were 6.07 mV, 5.91 mV and 5.75 mV, respectively (Figure 13a,c,e). This is due to the fact that the addition of SWMs and DWMs compared to SJ-0 slightly increased the internal pores of the mortar, which weakened the signal of ultrasonic wave propagation inside the mortar and eventually led to a decrease in the maximum amplitude of the main frequency. Compared with SWMs, DWMs have a larger particle size and a more pronounced effect on the mortar particle gradation, so the maximum amplitude of the main frequency of SJ-2 is relatively small. Figure 13b,d,f show that the maximum amplitudes of the main frequencies of SJ-0, SJ-1 and SJ-2 after self-repairing were 4.08 mV, 5.11 mV and 5.39 mV, respectively. The results indicate that the main frequencies of SJ-1 and SJ-2 have recovered substantially, especially the maximum amplitude of the main frequency of SJ-2 has been very close to the initial value, which indicates that the microcapsules are able to self-repair the defects such as microcracks and macropores inside the mortar. The results of FFT analysis are consistent with the ultrasonic waveform analysis in Section 3.9.

### 3.11. Variation of Surface Cracks Repairing Ratio

**Figure 14:**
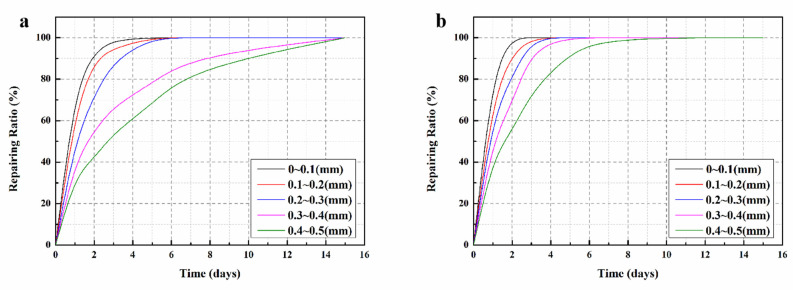
Surface cracks repairing ratios of mortars. (**a**) SJ-1, (**b**) SJ-2.

The variation of the surface crack repairing ratios with time for SJ-1 and SJ-2 are shown in Figure 14. The fitting results are displayed as Table 5. The fitted correlation coefficients (R^2^) were all greater than 0.9, indicating that the linear equation can accurately describe the self-repairing process of mortar surface cracks. From Figure 14a, it can be seen that the surface crack in SJ-1 with a width less than 0.3 mm can self-repair within 5 days, while the crack with a width more than 0.3 mm takes 15 days to completely self-repair. However, for surface cracks with width less than 0.1 mm in SJ-2 (Figure 14b), the self-repairing time is only 2 days in length, and surface cracks with width less than 0.3 mm can be completely self-repaired within 3 days. When the initial width of the surface crack reaches 0.4–0.5 mm, its self-repairing time is extended to 7 days. Comparing Figure 14a,b, it can be found that the self-repairing time of surface cracks in SJ-2 is significantly shorter than that of SJ-1 for the same initial width. This is because the shell material of DWMs added in SJ-2 contains Fe_3_O_4_ nano-particles with good magnetic permeability, which can generate heat under the action of external electromagnetic field and enable all microcapsules dispersed in the mortar to rupture in time. In addition, as that heat causes the temperature in the mortar to increase, it accelerates the reaction rate between the epoxy resin and 2-ethyl-4-methylimidazole, which improves the self-repairing efficiency of cracks in the mortar. The crack repairing ratio during the self-repairing process is counted, and the curve equation of the whole process is obtained by software simulation, so that the self-repairing of surface cracks can be predicted and monitored at any time, which is a good guideline for the engineering application of the microcapsule self-repairing method.

### 3.12. Visual Crack Closure

**Figure 15:**
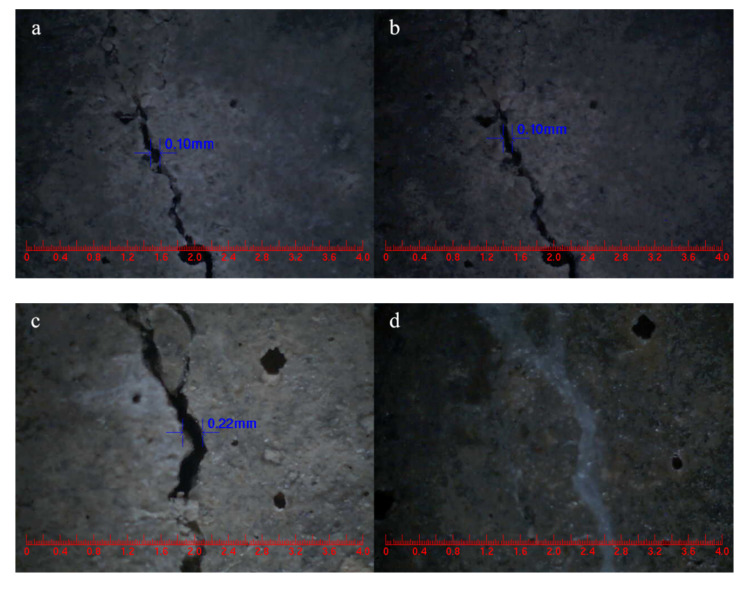

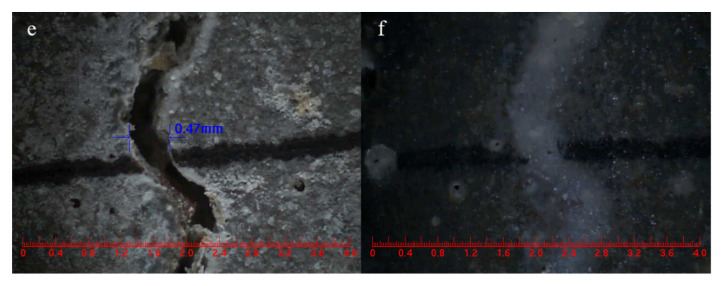
**Surface cracks width of mortars.** (**a**) SJ-0, (**b**) SJ-0 for 15 days self-repairing, (**c**) SJ-1, (**d**) SJ-1 for 15 days self-repairing, (**e**) SJ-2, (**f**) SJ-2 for 15 days self-repairing.

In this experiment, cracks were pre-fabricated on the surfaces of SJ-0, SJ-1 and SJ-2 by the three-point bending method, and their initial widths were measured. After 15 days of self-repairing, the crack widths were measured again, and the test results are shown in Figure 15. Observing Figure 15a,b, it can be seen that the width of SJ-0 with an initial width of 0.1 mm did not change before and after self-repairing, which indicates that the cementitious material cannot self-repair its surface cracks alone. However, the surface crack with an initial width of 0.22 mm in SJ-1 (Figure 15c,d) and 0.47 mm in SJ-2 (Figure 15e,f) can completely self-repair after 15 days. This indicates that the microcapsules can effectively self-repair the cracks in the mortar, and SJ-2 can self-repair cracks wider than SJ-1, which is consistent with Section 3.11.

## 4. Conclusions

In summary, new electromagnetic-induced rupture microcapsule (DWMs) of Fe_3_O_4_ nano-particles/polyethylene wax coated epoxy resin were successfully prepared by the melt dispersion method in this paper. The core fraction, compactness, particle size distribution, morphology, and chemical structure of the microcapsules were characterized. The microcapsules were then mixed into the mortar and the self-repairing ability of the mortar containing microcapsules was evaluated. The main conclusions are as follows:

(1) It is worth noting that the core fraction, compactness, and mean particle size of DWMs were significantly higher compared to the polyethylene wax coated epoxy resin microcapsules (SWMs), which contributed to the self-repairing ability of the microcapsules. The core fraction, remaining weight (30 days), and mean size of DWMs were 72.5%, 9.6 g and 220 μm, respectively.
(2) Scanning electron microscopy (SEM) showed that the DWMs were regular spherical with a rough surface, which can form a good bond with cement matrix. FTIR indicated that the epoxy resin was successfully encapsulated in the Fe_3_O_4_ nano-particles/polyethylene wax.
(3) After 15 days of self-repairing, the pre-damaged mortar showed 48.97%, 91.9%, and 24.03 mV of harmful pore ratio, compressive strength recovery, and maximum amplitude, respectively. Cracks with initial widths of 0.4–0.5 mm in the mortar were self-repaired within 7 days. The results showed that the mortar containing DWMs had excellent self-repairing ability.
(4) Based on the curve equations obtained from software simulations, the self-repairing of surface cracks can be predicted and monitored to calculate the crack repair ratio during the self-repairing process. This work is expected to provide new insights to address the mechanism of microcapsule rupture in self-repairing cement-based materials.

## Figures and Tables

**Table 1:** Preparation parameters of microcapsules.

Microcapsule	Shell Material Using by Mass Ratio	Heating Temperature	Stirring Rate	Core Material Using by Mass Ratio
SWMs	Polyethylene Wax, 40	125 °C	900 rpm	epoxy resin, 60
DWMs	Polyethylene Wax, 35; Fe_3_O_4_ nano-particles, 5	125 °C	900 rpm	epoxy resin, 60

**Table 2:** Mix designs of the mortars by the mass ratio.

Mortars	Cement	Sand	Water	Microcapsules
SJ-0	100	50	300	0
SJ-1	100	50	300	3 (SWMs)
SJ-2	100	50	300	3 (DWMs)

**Table 3:** Core fraction of SWMs and DWMs.

Microcapsule	Core Content
SWMs	64.9%
DWMs	72.5%

**Table 4:** Flexural strength and compressive strength of mortars.

Mortars	Flexural Strength	Compressive Strength
SJ-0	8.2 MPa	33.4 MPa
SJ-1	7.9 MPa	32.6 MPa
SJ-2	7.5 MPa	30.9 MPa

**Table 5:** Self-repair equations for surface cracks of all widths.

Mortars	Widths	Fitting Line	R^2^
SJ-1	0–0.1 mm	y = (98.26x^2^ + 198.6x + 99.52)/(x^2^ + 1.96x + 0.99)	0.9999
	0.1–0.2 mm	y = (99.22x^2^ + 190.9x + 91.67)/(x^2^ + 1.88x + 0.92)	0.9999
	0.2–0.3 mm	y = (84.73x^2^ + 315.4x + 218.6)/(x^2^ + 12.84x + 2.24)	0.9984
	0.3–0.4 mm	y = (1463x^2^ + 497,500x + 457,700)/(x^2^ + 4386x + 5776)	0.9965
	0.4–0.5 mm	y = (191,800x^2^ + 1,028,000x + 939,000)/(x^2^ + 8521x + 13,530)	0.9901
SJ-2	0–0.1 mm	y = (5722x + 5267)/(x^2^ + 55.16x + 52.77)	0.9945
	0.1–0.2 mm	y = (96.11x^2^ + 262.2x + 159.9)/(x^2^ + 2.524x + 1.604)	0.9913
	0.2–0.3 mm	y = (138,300x^2^ − 2,350,000x + 2,279,000)/(x^2^ + 20,640x + 22,840)	0.9913
	0.3–0.4 mm	y = (304,900x^2^ − 2,815,000x − 2,848,000)/(x^2^ − 22,230x − 28,920)	0.9861
	0.4–0.5 mm	y = (889x + 818.2)/(x^2^ + 6.463x + 9.078)	0.9945

## Data Availability

Not applicable.
